# BN-SNN: Spiking neural networks with bistable neurons for object detection

**DOI:** 10.1371/journal.pone.0327513

**Published:** 2025-07-10

**Authors:** Siddiqui Muhammad Yasir, Hyun Kim

**Affiliations:** Department of Electrical and Information Engineering, Research Center for Electrical and Information Technology, Seoul National University of Science and Technology, Seoul, South Korea; New York University Abu Dhabi, UNITED ARAB EMIRATES

## Abstract

Spiking neural networks (SNNs) are emerging as a promising evolution in neural network paradigms, offering an alternative to conventional convolutional neural networks (CNNs). One of the most effective methods for SNN development is the CNN-to-SNN conversion process. However, existing conversion techniques are hindered by long temporal durations or inference latencies, which negatively impact the accuracy of the converted networks. Additionally, the application of SNNs in object detection tasks remains largely under-explored. In this study, we propose a novel approach utilizing a bistable integrate-and-fire (BIF) neuron model integrated with a single-shot multibox detector (SSD) as the detection head. Leveraging the proposed BIF neuron framework, we convert the widely used ResNet architecture into an SNN. We validate the effectiveness of our approach through object detection tasks on the MS-COCO and Automotive GEN1 datasets. Experimental results show that our conversion technique facilitates object detection with reduced temporal steps and significant enhancements in mean average precision (mAP), achieving mAP@0.5 scores of 0.476 and 0.591 for the MS-COCO and Automotive GEN1 datasets, respectively. This research marks the first application of BIF neurons to object detection, presenting a novel advancement in the field.

## 1 Introduction

Deep neural network (DNN) architectures, particularly convolutional neural networks (CNNs), have established new benchmarks in computer vision tasks, achieving state-of-the-art (SOTA) performance in object detection. However, developing deep learning systems presents several critical challenges. A major issue is the emphasis on enhancing computational power and performance, often at the expense of energy consumption. Recently, the advantages of low-energy artificial intelligence have gained significant attention from researchers, driven by both cost-efficiency and environmental benefits [1].

In recent years, researchers have developed compression algorithms (*e.g.*, pruning [2–4], quantization [5–7], and compression [8–10]) to significantly reduce the network parameters and computational demands of DNNs while preserving their performance. Concurrently, efforts are focused on optimizing computing architectures [11] to lower computational energy consumption by designing hardware that better aligns with DNN model features [12–16]. Despite these advancements, the challenge of high computational complexity inherent in DNNs persists [1,11,12,17].

To address these limitations, recent research has focused on spiking neural networks (SNNs), which operate in an event-based manner, thereby reducing both latency and computational load [18–22]. Unlike CNNs, SNNs can produce results almost immediately after the first output spike, making them highly suitable for real-time applications [23]. Furthermore, SNNs are naturally adept at processing input from event-based sensors [24] and have demonstrated high efficiency and accuracy in traditional frame-based computer vision tasks, such as object recognition and detection, especially when implemented on neuromorphic hardware [25]. While multi-layered spiking networks have been successfully implemented on platforms such as FPGAs [26,27], more extensive networks comprising tens of thousands of neurons have been deployed on large-scale neuromorphic platforms [25]. These platforms have demonstrated the capability to implement CNNs with over a million neurons while maintaining minimal power dissipation, thus paving the way for energy-efficient deep learning [28]. The ability to map deep CNN architectures, like VGG-16, onto these platforms without compromising performance opens the door to deploying deep spiking networks in real-world applications [25,26,29,30]. To bridge the gap between continuous-valued deep learning networks and neuromorphic spiking networks, several methods have been developed to achieve comparable error rates with SNNs. These techniques include direct training using backpropagation [31], stochastic gradient descent for classifier layers [32], and modifying CNN transfer functions to improve parameter mapping to SNNs [33]. Despite promising results, these methods are not yet mature enough to train spiking architectures at the scale of VGG-16 and achieve error rates comparable to their CNN counterparts. A more straightforward approach involves converting pre-trained CNNs to equivalent SNNs. This method, initially explored by [34] in 2013 and recently refined by others [35], aims to retain CNN performance while leveraging the efficiency of SNNs. Despite successes with simpler tasks like MNIST, scaling to more complex datasets like CIFAR-10 [36,37] and ImageNet [38] remains challenging owing to the lack of spiking equivalents for essential CNN operations, such as max pooling and batch normalization [39].

In this work, we propose using bistable neural networks (BSNNs) as an innovative approach to address challenges in object detection tasks. Bistability, a phenomenon observed in biological neurons by Izhikevich [40] in 2003, enables neurons to switch between spiking and non-spiking states, facilitating short-term memory functions [41,42]. By integrating bistability into the network architecture, we aim to enhance the performance and efficiency of deep spiking networks. We introduce a BSNN framework as a feature extractor for object detection, which leverages phase encoding and bistability to significantly improve performance after converting from CNNs, while also reducing latency. For high-performance Spiking ResNet implementations, synchronous neurons are utilized to ensure coherent spike propagation through residual blocks. This approach significantly reduces the number of time steps needed to achieve optimal performance, paving the way for the practical deployment of BSNNs in real-world object detection scenarios. This research aims to develop a robust and energy-efficient neural network backbone for detection tasks by advancing the integration of bistable mechanisms into spiking networks. This approach addresses both computational efficiency and performance requirements in modern computer vision applications. The contributions of this study are summarized as follows:

- A novel architecture for object detection tasks is proposed, incorporating bistable neurons that combine phase encoding and bistability mechanisms as the foundation for the detection head.
- BIF neurons are integrated with ResNet, endowing the network with specific capabilities for object detection.
- The architecture is enhanced with the detection head of an SSD [43], enabling effective utilization for object detection applications.
- Empirical results demonstrate that the proposed method achieves object detection with reduced temporal intervals and significantly improves mean average precision (mAP).

The structure of this paper is organized as follows: Sect 2 provides a comprehensive review of related works, situating this study within the broader context of prior research. Sect 3 introduces integrate-and-fire (IF) neurons, elaborates on the encoding techniques employed in this study, and details the proposed feature extraction method developed specifically for the detection head of the neural network. Sect 4 presents the experimental results of the proposed network, evaluated on the MS-COCO and Automotive GEN1 datasets, highlighting its performance and effectiveness. Finally, Sect 5 concludes the paper by summarizing the key findings and outlining potential directions for future work.

## 2 Related works

Recent research has presented several significant approaches and designs for converting CNNs to SNNs, including phase encoding, synchronous neurons, Spiking ResNet, and the spike-induced norm activation function. Spiking neural networks (SNNs) have emerged as energy-efficient alternatives to traditional convolutional neural networks (CNNs), with recent advancements focusing on leveraging SNNs for complex tasks such as object detection. A significant body of research has explored methods to bridge the performance gap between SNNs and CNNs, often through hybrid architectures or conversion techniques.

One widely adopted approach involves converting CNNs into SNNs to achieve accuracy comparable to their CNN counterparts [44]. This process typically entails training a CNN using backpropagation and subsequently mapping its activations to spiking neurons. Techniques such as quantization-aware training, establishing a one-to-one correspondence between CNN and SNN neurons [11,45], and setting appropriate firing thresholds [46] have been proposed to facilitate this transformation. These conversion methods ensure that spiking neurons approximate the activation patterns of ReLU units in CNNs, thereby preserving task-specific performance metrics. Recent innovations, such as Spiking ResNet architectures, incorporate spiking neurons (e.g., integrate-and-fire (IF) and leaky integrate-and-fire (LIF) neurons) into residual blocks to enhance performance on image recognition tasks [47]. These architectures demonstrate the functional similarity between ReLU and IF neurons, leveraging this resemblance to simplify conversion processes. In addition, layer-wise adjustments of SNN parameters based on CNN activation values have been shown to improve temporal efficiency and overall accuracy [48].

While CNN-to-SNN conversion remains a dominant approach, direct training of SNNs has gained traction, especially for tasks involving object recognition and detection. Directly trained SNNs leverage event-driven data to exploit their inherent temporal and energy efficiency advantages [49]. Supervised learning in SNNs commonly employs surrogate gradient techniques to overcome the non-differentiable nature of spike-based activations, enabling applications of backpropagation [50]. Alternatively, unsupervised learning methods, such as spike-timing-dependent plasticity (STDP), offer biologically inspired training paradigms but often underperform in comparison to supervised methods [51].

Hybrid architectures that integrate CNNs and SNNs have also emerged as a promising direction. These architectures typically use a CNN backbone for feature extraction while employing SNNs to process temporal dynamics, achieving a balance between accuracy and energy efficiency [52]. For example, hybrid networks have been applied in event-based vision tasks, where SNNs handle asynchronous spiking data, and CNNs process synchronous visual features for classification and detection. These approaches leverage the complementary strengths of both networks, resulting in improved performance in tasks such as image classification and object detection [17,53,54].

A relatively underexplored area involves the use of bistable neurons in SNNs. Bistable neurons exhibit two stable states, enabling robust temporal coding and improving feature extraction capabilities for dynamic tasks like object detection. These neurons allow for efficient integration of temporal and spatial information, making them particularly suitable for event-based processing. Although much of the existing research has focused on simpler neuron models, such as IF and LIF neurons, bistable neurons offer promising potential for advancing SNN applications in object detection.

Despite significant advancements in SNN architectures and training methods, most studies have concentrated on image classification tasks with shallow networks and datasets like MNIST and CIFAR [37,55]. Object detection, a more complex task, requires efficient feature extraction and spatiotemporal integration, making it an ideal application for SNNs. Recent works have demonstrated that event-based datasets such as MS-COCO and Automotive GEN1 can be effectively processed using SNNs, highlighting their suitability for real-world scenarios [36,49]. Techniques incorporating bistable neurons into detection pipelines could further enhance the temporal resolution and energy efficiency of SNNs in these tasks.

In summary, while CNN-to-SNN conversion, direct training, and hybrid networks have significantly contributed to the development of SNNs, the integration of bistable neurons presents a novel and promising approach for object detection tasks. Future research should explore these mechanisms in greater depth to fully exploit the advantages of SNNs for dynamic and energy-efficient applications. The research in the field of CNN-to-SNN conversion is focused on developing effective methodologies for seamlessly transitioning from traditional CNNs to SNNs. The goal is to maintain accuracy, leverage the strengths of both architectures, and optimize the conversion process for various applications.

## 3 Methodology of CNN-to-SNN conversion

This section details the core process of converting CNNs to SNNs. It includes an explanation of the BIF neurons and encoding methods used, followed by the integration of an SSD detection head within the conversion framework. The impact of synchronized neurons in Spiking ResNet architectures is discussed, and a thorough outline of the proposed model architecture is provided to address time delays and conversion losses.

### 3.1 Spiking integrated-and-fire neuron encoding

The IF model is a widely used spiking neuron model in neural networks. In this framework, a neuron accumulates input spikes from presynaptic neurons, which dynamically modulate its membrane potential. When the membrane potential reaches a predefined threshold, the neuron generates an action potential and typically resets its membrane potential to zero, potentially leading to the loss of critical information [56].

To address this issue, our methodology introduces a novel conversion technique from analog to SNNs. As proposed by [42], this technique incorporates a modification that subtracts the threshold value from the membrane potential. This modification aims to reduce the information loss associated with the traditional reset mechanism inherent in the IF model [57].

$$V_{i,t}^{\left(l\right)}=V_{i,t-1}^{\left(l\right)}+\sum_{j}w_{ij}\delta_{j,t}^{\left(l-1\right)}$$ (1)

where *w*_*ij*_ represents the weight connecting the neuron *j* to neuron *i*, and $\delta_{j,t}^{\left(l-1\right)}$ denotes the spike of neuron *j* in layer (*l*–1) at time *t*. $V_{i,t}^{\left(l\right)}$ denotes the membrane potential of neuron *i* in layer *l* at time *t*.

The firing dynamics of neurons can be quantitatively characterized using real-valued representations via various encoding schemes (refer to Eq (1)). Spike trains can be represented as real numbers using several approaches. In rate coding (*a*_*rate*_), the real value is associated with the firing rate, which is defined as the frequency of spikes occurring over a specified time interval. Conversely, in temporal coding (*a*_*temporal*_), the real value is derived from the time difference between the total simulation time *T* and the occurrence of the spike time *t*_*spike*_. In this framework, *N* denotes the total number of spikes, and *t*_*spike*_ represents the timestamp of the initial spike.

Empirical evidence from prior studies has highlighted that both rate and temporal coding schemes are subject to considerable temporal delays. As delineated in Eq (2), these encoding methods generally necessitate a minimum of 1,000 time steps to accurately encode a minimal input value of 0.001.

$$a_{rate}=\frac{N}{T}\;\text{and}\;a_{temporal}=1-\frac{t_{spike}}{T}$$ (2)

Consequently, phase coding [58] has been employed to encode activation values into spike trains. This method enhances the information density per spike by assigning distinct weights and thresholds to each phase, thereby optimizing the representation of activation levels. As a result, phase coding exhibits superior energy efficiency. Empirical evaluations demonstrate that phase coding provides a more precise and efficient representation of the activation values compared to alternative encoding schemes.

$$a_{j}^{l}=\frac{1}{n}\sum_{k=1}^{nK}S_{k}\delta_{j,k}^{l}$$ (3)

where $a_{j}^{l}$ denotes the activation value of neuron *j* in layer *l*. *K* represents the phase count within a period. $n=\frac{T}{K}$ defines the number of period. The phase function *S* defined as:

$$S_{t}=2^{-\left(1+mod(t,K)\right)}$$ (4)

To address the significant temporal latency inherent in converted SNNs, the technique of weighted spiking has been employed. This method assigns varying weights to spikes at different phases, thereby encoding additional information within each spike [58]. However, deviations occur when neurons fail to spike within the anticipated temporal phase, leading to discrepancies in spike encoding among neurons in hidden layers. Such deviations can impair performance, particularly in complex datasets and large-scale network architectures. To compensate for a deficit in spike count within a given time step (0,*t*), neurons are subjected to augmented current later in the process to increase spike frequency. However, if a neuron’s activation value remains sub-threshold, unstable synaptic currents can inadvertently trigger spikes once the threshold potential is surpassed, resulting in spikes from inactivated neurons (SIN). Consequently, the SNN must accumulate spikes over extended periods to mitigate the impact of these erroneous spikes, ensuring that the network’s firing rates align with the feature representations of CNNs. This need for prolonged accumulation contributes to the significant time delays observed in converted SNNs. In scenarios where SIN is pronounced—such as when numerous non-activated CNN features are erroneously triggered in the SNN—extended simulation periods and rate-based conversion methods fail to resolve the issue, leading to notable performance degradation.

### 3.2 Bistable integrated-and-fire neuron

The immediate neuronal response to incoming current can exhibit inherent unreliability, potentially causing stability issues in SNNs, particularly those related to phase lead and phase lag. To address these issues, it is crucial to ensure that information propagates consistently across spiking neurons, thereby adhering to encoding protocols.

To address these challenges, integrating the BIF neuron model with a bistability mechanism is adopted. This approach employs a piecewise function to represent spiking dynamics, capturing the periodic alternation between spike and non-spike states inherent to bistability. In this framework, neurons display spiking activity in response to membrane potential changes during the spike phase and remain inactive during the non-spike phase. By aligning neuronal behavior with the bistability characteristics, this method ensures more reliable and stable spike train encoding.

$$\delta_{X,i,t}^{l}={\{}_{\text{0,}\;\text{otherwise}}^{H(V_{X,i,t}^{l}-V_{\text{th},t}),\;\text{if mod}\left(\left\lfloor \frac{t}{K}\right\rfloor ,2\right)=1,}$$ (5)

In Eq (5), $V_{X,i,t}^{l}$ represents the membrane potential of neuron *ith* in layer *l* at time step *t* under the input *X*. This value accumulates presynaptic input over time and does not directly correspond to spike output. The spike output is represented by *S*_*i*,*t*_ where Eq (6) ensuring that neurons fire only when their membrane potential surpasses the threshold. Spike generation occurs only when the membrane potential surpasses the firing threshold $V_{th}$. The spike output *S*_*i*,*t*_ is a discrete event and is formally governed by the Heaviside step function:

$$S_{i,t}=H(V_{X,i,t}-V_{\text{th},t})$$ (6)

This ensures that neurons alternate between accumulation and firing phases, preventing unnecessary activations and improving efficiency in object detection tasks.

It is a binary indicator function that determines whether a neuron fires a spike (δ=1) or remains silent (δ=0)based on its membrane potential and a phase-dependent condition. Traditional SNNs may suffer from unstable or excessive spiking, leading to inefficient computation. “δ” introduces temporal regulation, reducing redundant spikes and making SNNs more energy-efficient. “δ” acts as a mechanism that regulates neuron spiking by combining threshold-based activation with a bistable switching condition. Let *H*(*x*) denote the unit step function and $\left\lfloor \frac{t}{K}\right\rfloor$ which represent the floor function. In the context of periodic input, neurons are not required to continuously respond to incoming spikes. Instead, they accumulate spikes and respond to the loop. The neuron produces a spike based on the following conditions: Firstly, whether the membrane potential $V_{X,i,t}^{l}$ exceeds a threshold $V_{\text{th},t}$ and secondly, whether the system is in a specific phase (controlled by the periodic condition $mod\left(\left\lfloor \frac{t}{K}\right\rfloor ,2\right)=1$. The *K* defines the number of phases in the phase-coding mechanism, and the time window size significantly influences the temporal dynamics and accuracy of the BIF-based SNN. A larger *K* increases the information density per spike but may lead to phase synchronization issues in large networks.

$V_{X,i,t}^{l}$ represents the membrane potential of a neuron in layer *l*, indexed by *i*, at time step *t*, within a system or network associated with input or state *X* in Eq (5). The Heaviside step function *H*(*x*) defined as: H(x)=1 if x≥0, and H(x)=0 otherwise. Evaluate if the neuron is eligible to fire whether the membrane potential $V_{X,i,t}^{l}$ has crossed the threshold $V_{\text{th},t}$. During the non-spike phase, neurons accumulate spikes, enabling precise responses during the active phase and reducing issues with phase lead or lag. This process is illustrated in Eq (5) by the connections between units *X*, which are alternately represented by neuron *A* as: $H(V_{A,i,t}^{l}$ − $V_{\text{th},t})$ or neuron *B* as: $H(V_{B,i,t}^{l}$ − $V_{\text{th},t})$. Specifically, neurons with identical synaptic weights, such as neuron *A* in one unit, are coupled with neuron *B* in another unit. The postsynaptic neuron, during its non-spike phase, collects spikes throughout the spike phase of the presynaptic neuron, ensuring accurate responses in the subsequent active phase.

The $mod\left(\left\lfloor \frac{t}{K}\right\rfloor ,2\right)=1$ defines a bistable temporal gating condition. Where, $\left\lfloor \frac{t}{K}\right\rfloor$ divides the time *t* into blocks of length *K* and rounds down to the nearest integer. Alternates between 0 and 1 across consecutive *K*–*length* blocks. Which ensures the neuron can fire only during every other *K*–*length* block of time. This bistable condition introduces phase-dependent behavior, allowing the neuron to alternate between active and inactive states. The 0, otherwise ensures that if the neuron is in the “inactive" phase or $V_{X,i,t}^{l}$ doesn’t exceed $V_{\text{th},t}$ it remains silent (i.e., $V_{X,i,t}^{l}=0$).

The selection of T = 1000 as the temporal window size in our BSNN framework is based on theoretical encoding constraints and optimal neuron firing strategies. As described in Eq (2), at least 1000 time steps are required to accurately encode minimal input values (0.001) in rate-based and temporal coding schemes. This ensures a sufficient accumulation period for neurons before firing, allowing reliable feature retention while avoiding uncontrolled spike generation. Additionally, the phase coding mechanism (Eqs (3) and (4)) enhances spike modulation by distributing activations over alternating firing phases. This structured encoding process prevents redundant activations and ensures stability, reinforcing why T = 1000 balances accuracy, energy efficiency, and computational overhead for object detection tasks.

By combining membrane potential dynamics with phase-dependent gating, bistable integrated-and-fire neurons achieve both selectivity and temporal modulation of activity. To encapsulate one activation value in the CNN, a pair of BIF neurons been employed as a single computational unit, formalized as follows:

$$\delta_{Xi,t}^{l}=\delta_{B,i,t}^{l}\;or\;\delta_{A,i,t}^{l}$$ (7)

The rationale for employing a pair of BIF neurons in this framework stems from the observation that individual BIF neurons exhibit non-spiking periods for approximately half of the simulation time. Utilizing two neurons with complementary spike states ensures that information is effectively transmitted to the subsequent layer, maintaining the continuity of information flow. Specifically, one neuron within each adjacent layer remains in a spiking state to release stored memory, while the other remains in a non-spiking state to handle incoming spikes. This configuration helps mitigate the potential disruption caused by a non-spiking neuron in the previous layer, as its inactivity does not interfere with the spike transmission to the subsequent layer.

Furthermore, employing two BIF neurons improves the scalability of the network. This design allows the conversion of CNNs with various topologies without the need to meticulously design the spiking stage for each layer, even when transitioning to deeper and wider CNNs [42]. Using only a single BIF neuron per layer would limit functionality owing to its inability to effectively accumulate spikes, as described.

The rationale behind employing a pair of BIF neurons in a single computational unit stems from their bistable dynamics, which enhance information transmission and stability. BIF neurons alternate between two stable states, spiking and non-spiking, providing distinct advantages in signal processing. A single BIF neuron, while efficient, remains inactive for approximately half of its operating time. This introduces potential latency and discontinuity in information flow, particularly in networks requiring high-throughput synchronized data transfer across layers. By incorporating two BIF neurons with complementary spiking phases in a single cell, the following theoretical benefits are realized.

**Continuous Information Flow:** A single BIF neuron alternates between spiking and non-spiking states, creating gaps in the signal propagation. With two neurons operating in complementary phases, at least one neuron is always active, ensuring uninterrupted signal transmission. Which is mathematically formalized as in Eq (7), where $\delta_{A,i,t}^{l}$ and $\delta_{B,i,t}^{l}$ denote the outputs of the two BIF neurons in layer *l*, indexed by *i*, at time step *t*. Their complementary nature guarantees that $\delta_{i,t}^{l}$ remains non-zero for every *t*, facilitating consistent information flow. This improves temporal memory, stability, and computational efficiency in BIF neurons.

**Enhanced Temporal Stability:** The bistable design inherently reduces phase synchronization issues within the network. A single BIF neuron is vulnerable to phase misalignment, especially in deep architectures where phase lag accumulates. Complementary neurons mitigate this risk by allowing the inactive neuron to accumulate spikes during the other’s spiking phase. This mechanism not only reduces latency but also prevents information loss caused by phase discrepancies.

**Improved Robustness to Synaptic Noise:** In neuromorphic systems, synaptic noise or variability can cause misfiring or signal corruption. The dual-neuron configuration leverages redundancy, ensuring that even if one neuron experiences a perturbation, the complementary neuron maintains functional integrity. This design enhances the reliability of spike-based computations in noisy environments.

**Scalability for Deeper Architectures:** When extending spiking neural networks to deeper and wider architectures, the dual-neuron setup simplifies the conversion process by maintaining robust spike propagation across layers. Without this dual arrangement, single BIF neurons may not accumulate spikes adequately, particularly in layers that require complex feature extraction.

**Energy-Efficient Encoding:** Although two neurons are utilized, the complementary nature of their operation ensures that the overall firing rate is distributed, not doubled. This balanced activation pattern reduces the likelihood of energy-intensive, simultaneous firing across all neurons in the network. Furthermore, the bistability mechanism optimizes spike utilization by encoding more information per spike.

Moreover, integrating bistable neurons with phase encoding enhances information transmission within the network through an accumulation-and-firing process in each layer. During the accumulation phase, the information is precisely transmitted to the subsequent layer with a one-cycle delay that reduces the immediate impact of synaptic currents on the conversion process. This approach improves the robustness and precision of the network output.

### 3.3 Spiking ResNet

**Fig 1:**
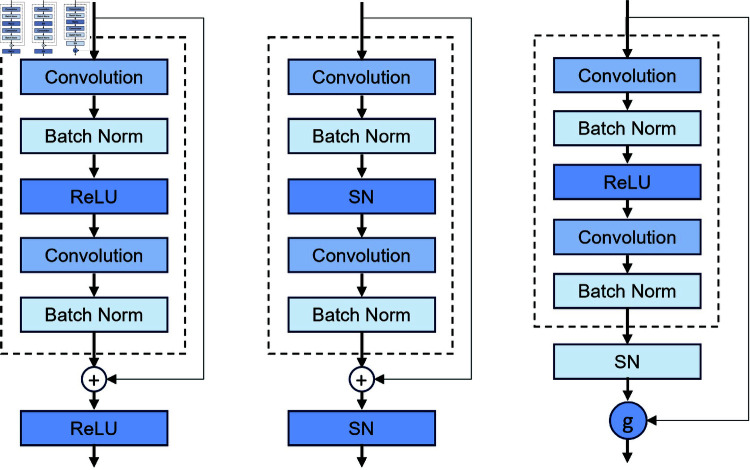
Description of residual blocks for ResNet, Spiking ResNet, and SEW-ResNet.

The residual block represents the fundamental building block of the ResNet architecture. As illustrated in Fig 1, the basic ResNet block [59,60] consists of an input *X*_*l*_ and output *Y*_*l*_ for the *l*–*th* layer block, which “Conv," “BN," and “ReLU" representing the convolutional layer, batch normalization, and rectified linear unit activation layer, respectively. In Spiking ResNet [60,61], the traditional residual block is modified by substituting ReLU activation layers with spiking neurons (*i.e.*, “SN"), as depicted in Fig 1. Here, $S_{l}[t]$ and $O_{l}[t]$ denote the input and output of the *l*–*th* block in Spiking ResNet at the time step *t*, respectively. Despite this adaptation, Spiking ResNet does not universally apply to all neuron models for effective identity mapping, which may limit its performance and generalization across various neural network applications.

A fundamental concept in ResNet architectures is identity mapping, as discussed by [62]. They proposed that if additional layers in a deep network perform identity mapping, the training error of the deeper model should not exceed that of its shallower counterpart. However, achieving effective identity mapping within additional layers in a practical training timeframe remains challenging. This difficulty leads to the degradation problem, where deeper models exhibit inferior performance compared to shallower ones.

SEW-ResNet [63] and EMS-Yolo [64] have advanced SNN architectures to over 100 layers. SEW-ResNet, operates primarily through integer multiplication within the residual network framework, whereas EMS-Yolo focuses on the spiking dynamics of residual paths but neglects the non-spiking convolutions in shortcut paths. This neglect is particularly problematic in object detection tasks, where the varying dimensionality and channel counts can exacerbate energy inefficiencies associated with non-spiking convolutions.

The limitations present in SEW-ResNet and EMS-Yolo can be effectively addressed by employing BIF neurons. BIF neurons offer a more efficient method for maintaining identity mapping in deeper network architectures. Their bistable nature enhances the stability and consistency of signal propagation through layers, thus mitigating the degradation problem. Additionally, BIF neurons improve energy efficiency by reducing the computational load associated with non-spiking convolutions in shortcut paths. This approach not only preserves the integrity of identity mapping but also significantly reduces energy consumption, making it a valuable solution for scaling SNNs to greater depths and complexities [65], especially in large-scale object detection tasks.

### 3.4 Residual block based on bistable IF neurons

To effectively operationalize SNNs, preprocessing CNNs before conversion is crucial. Given that SNNs have a maximum firing rate of one—owing to the fact that neurons can emit only one spike per time step—a data normalization method is employed. This method, detailed in Eq (8), is used to normalize the weights and biases accordingly.

$${\hat{w}}_{ij}^{l}=\frac{w_{ij}^{l}\lambda^{l-1}}{\lambda^{l}}\;\text{and}\;{\hat{b}}_{i}^{l}=\frac{b_{i}^{l}}{\lambda^{l}}$$ (8)

In this context, ${\hat{w}}_{ij}^{l}$ and ${\hat{b}}_{i}^{l}$ represent the weights and biases used in the SNN, while $\lambda_{l}$ indicates the maximum activation value for the *l*–*th* layer. This method ensures that each activation value in the CNN is constrained to a maximum of 1. Max pooling and BN operations are performed using the spike from the neuron with the highest firing rate as the output, as described in Eq (9). During training, the BN layer and block convolution layer are integrated to convert the residual block into its corresponding SNN version. The parameters of the new convolutional layer, which can be converted, are defined as follows:

$${\hat{w}}_{ij}=\frac{\gamma_{i}}{\theta_{i}}w_{ij}\;\text{and}\;{\hat{b}}_{i}=\frac{\gamma_{i}}{\theta_{i}}(b_{i}-\mu_{i})+\beta_{i}$$ (9)

where *x* represents the input to the BN layer, given by $BN[x]=\frac{\gamma }{\theta }(x-\mu )$. Here, μ and θ denote the mean and variance of the batch, respectively, and β and γ are the learned parameters during training.

**Fig 2:**
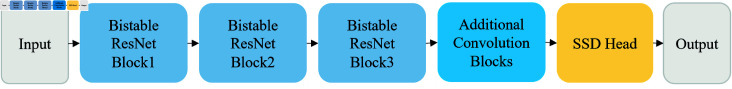
Conceptual overview of proposed spiking ResNet + SSD architecture for object detection.

The ResNet architecture Fig 2 employs a residual block design that integrates dual information pathways: the shortcut connection either bypasses the convolutional layers to propagate the input to the output directly or integrates it via a convolutional operation. To optimize the conversion procedure, the convolutional and BN layers are fused into a unified computational module. During this conversion process, two fundamental challenges must be addressed.

The asynchronous scaling of information between the two pathways in a residual block poses significant challenges. Specifically, the data conveyed by the two paths to the output neurons are not proportionally aligned with the activation values because of the difficulty in normalizing the shortcut path, which lacks convolutional layers. The shortcut path has one fewer ReLU operation compared to the convolutional path, resulting in a difference of two BIF neurons in the SNN. This discrepancy occurs because neurons must accumulate membrane potential before firing, leading the information from the shortcut path to reach the output neuron more quickly than from the convolutional path. To address this issue, scale parameters are calibrated based on the maximal activation values of both input and output, ensuring that the combined information from both routes at the output neuron is proportionate to the activation levels Eq (10). SNNs are sensitive to temporal mismatches, and the neurons receive spikes from both the shortcut and convolutional paths. If these paths have different firing rates, phase mismatches can occur, leading to instability. The scaling factor ensures that spike rates are balanced, allowing information to reach the output neuron simultaneously.

**Fig 3:**
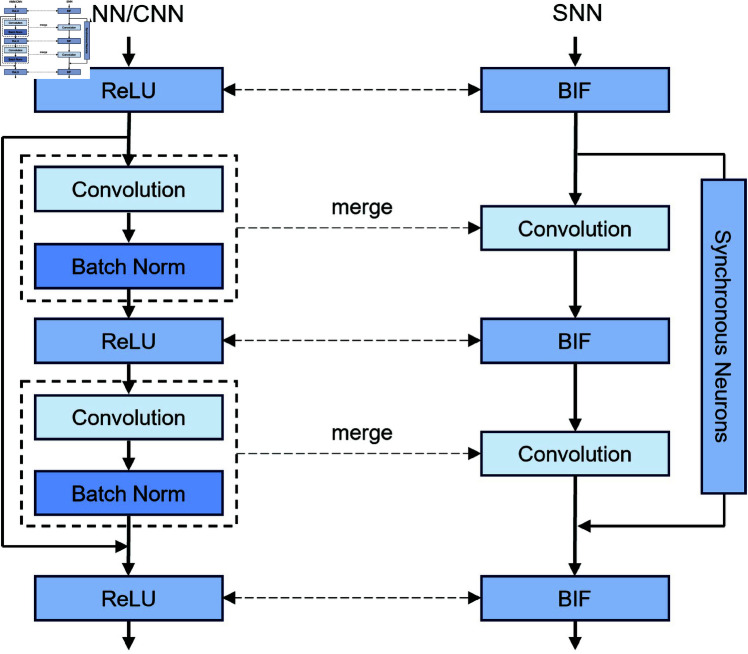
Synchronous neurons in the proposed backbone (Bistable ResNet Block) of Spiking ResNet.

Incorporating synchronous neurons—specifically with BIF neurons—into the shortcut path is a key consideration for this approach. This addition effectively simulates the inclusion of a ReLU function in the shortcut path of CNNs. As illustrated in Figs 1 and 3, this modification ensures synchronous transmission of information through the residual block. Given that the input to the shortcut path is non-negative, the impact on CNN transmission is minimal. In SNNs, the synchronous neurons ensure that information from both the shortcut and convolutional paths reaches the output neuron simultaneously. This approach addresses issues related to phase discrepancies and SNN instability within Spiking ResNet architectures.

$$scale=\frac{\lambda_{in}}{\lambda_{out}}$$ (10)

The scaling factor plays an important role in balancing spike flow by ensuring that the input and output firing rates are proportionally aligned. In Eq (10), the input firing rate $\lambda_{in}$ and the output firing rate $\lambda_{out}$ represents the number of spikes per unit time entering a neuron or layer. The *scale* is the ratio between $\lambda_{in}$ and $\lambda_{out}$ ensures the proper spike flow in the network. Eq (10) defines a scaling factor that adjusts the balance between input and output firing rates in a spiking neural network (SNN), particularly for Spiking ResNet architectures. The scaling factor regulates the firing rate, preventing excessive or insufficient spike activity, harmonizes spike timing, and reduces phase discrepancies and accuracy. Fig 1 illustrates the conversion mechanism for residual blocks. Information propagates to the output of the residual block via synchronous neurons. Given that the input to the shortcut path is entirely non-negative, the transmission within the CNN framework remains unaffected.

Fig 3 illustrates spike-based spike transmission using bistable neurons. The membrane potential $V_{X,i,t}$ accumulates charge, determining whether a neuron should fire a spike. Instead of continuous firing, neurons utilize a bistability mechanism, alternating between active and inactive phases. The firing condition follows a threshold-based activation function, ensuring regulated spike propagation. The binary spike output is formally represented in Eq (6). This design minimizes unnecessary activations, optimizing detection efficiency in Spiking ResNet architectures. In SNNs, however, the presence of synchronous neurons ensures that outputs from both the shortcut path and the convolutional path converge at the output neuron simultaneously.

This synchronization effectively mitigates phase lead and lag issues, as well as SNN instability problems, within the Spiking ResNet architecture. Motivated by advancements in CNNs, we have engineered SNNs utilizing exclusively strided convolutions, max pooling, BN, and Poisson BIF neurons. Strided convolutions and max pooling have been widely applied in the context of SNNs [66,67]. Notably, batch normalization (BN) layers can be integrated with preceding or succeeding convolutional layers during inference and are applicable for training SNNs when positioned prior to the BIF neurons. The significance of these layers is explored in Sect 3.4. In traditional CNNs, the final layers responsible for classification must be adapted to accommodate spike-based data. To address this, we introduce a spiking classifier that consists of a batch normalization (BN) layer, a 1×1 convolution layer producing class-specific channels, and BIF neurons. This classifier uses a 1*D* convolutional layer to manage feature maps of arbitrary dimensions, eliminating the need for average pooling layers, which are incompatible with spiking operations. The output spikes are first aggregated across the spatial dimensions and then across the temporal dimensions to generate predictions.

### 3.5 Detection head fusion with bistable residual blocks

The spiking fusion module integrates and refines the feature maps from both the backbone network and the Extra Block, subsequently feeding these enhanced feature maps into the SSD’s detection head. In object detection, the SSD framework [43] is renowned for its precision and efficiency. The SSD architecture typically consists of a backbone network, such as VGG16, paired with a detection head, making it highly adept at performing general object detection tasks [68,69]. Various enhancements have been proposed to improve SSD’s performance, particularly in detecting small objects. These include feature fusion, multiscale feature map skip connections, and trident features [70].

The SSD framework is distinguished by its use of a backbone network and multiple prediction heads, which exploit feature maps generated at various scales by the backbone to predict bounding boxes and their corresponding class labels. To adapt this framework within BSNNs, we substituted the conventional CNN backbone with BSNN backbones designed for classification, incorporating bistable convolutions in the supplementary layers. Consequently, the feature maps input to the SSD heads are bistable signals. Given that the heads consist solely of a single convolutional layer, the entire network operates as a BSNN. Over a temporal window of *T* timesteps, final bounding boxes, and class predictions are computed by aggregating these outputs across these timesteps. Although post-processing is required to filter predictions, this step is assumed to be performed on traditional hardware external to the bistable neural network.

Several critical modifications were made to integrate the SSD framework with a modified ResNet architecture that incorporates BIF neurons. The standard convolutions in the ResNet backbone were replaced with BIF convolutional layers, and BN layers were substituted with BIF ReLU activations. Specifically, each convolutional block now comprises a 1×1 BIF convolution for channel reduction, followed by a 3×3 BIF convolution with a stride of 2. Subsequently, BN layers are replaced by a second BIF convolution, and the final ReLU activation in each block is replaced by a BIF neuron. Additionally, scale and synchronous neurons were introduced to enhance the temporal stability and synchronization of the bistable signals.

One-shot object detectors, such as SSD, encounter challenges related to class imbalance owing to the dominance of background classifications. To address this, [71] introduced focal loss, which applies a modulation factor to the cross-entropy loss function, significantly enhancing the learning process for one-shot object detectors. Consequently, we train our bistable neural network using focal loss because the traditional hard negative mining approach employed by SSD did not yield satisfactory results. Consistent with the original SSD architecture, we incorporated three additional convolutional blocks to produce smaller feature maps from our bistable backbone. Each block comprises a 1×1 BIF convolution for channel reduction, followed by a 3×3 BIF convolution with a stride of 2. Anchors were generated with a minimum ratio of 0.5 and a maximum ratio of 0.8 to better capture smaller objects in the dataset. The architecture of our bistable ResNet34 + SSD system is illustrated in Fig 3, where progressively smaller feature maps from the ResNet backbone are fed into the SSD heads, and the three additional blocks further reduce the feature maps to a final dimension of 2×1.

This modified SSD head architecture ensures that the network fully leverages the bistable properties of the integrated BIF neurons, enhancing both the spatial and temporal resolution of the detection process. The integration of scale and synchronous neurons further stabilizes the network and ensures synchronization across different layers, thereby optimizing detection performance in dynamic environments.

## 4 Evaluation

### 4.1 Dataset description

Object detection is crucial in numerous applications, including self-driving cars and scene or object analysis. However, acquiring high-quality datasets for such tasks can be challenging. In this section, we utilize the Microsoft Common Objects in Context (MS-COCO) [72] and Automotive Gen1 [73] datasets to evaluate and compare our proposed approach. The MS-COCO dataset features numerous objects in different environments, while the Automotive Gen1 focuses on different car-related scenes. The MS-COCO dataset serves as a comprehensive benchmark for object detection, segmentation, and captioning tasks. It comprises 223,000 images across 81 object categories, divided into 118,000 training and 5,000 testing images. In this study, models were trained using data augmentation techniques and evaluated on the COCO validation set to detect 80 objects. The dataset’s diversity and complexity make it well-suited for developing models with extensive object recognition capabilities. Meanwhile, the largest event-based dataset is the Automotive GEN1 Detection dataset, which includes 39 h of recordings from an Automotive GEN1 sensor installed on the car’s dashboard. This dataset contains more than 255 K manually annotated bounding boxes, including those of cars and pedestrians. Because of the availability of several frames per second, this data is particularly effective for automotive systems used in detecting dynamic scenes. Furthermore, the GEN1 Automotive Detection dataset serves as the foundation for a new dataset called GEN1 Automotive Classification. It focuses on classifying samples captured from a car and includes bounding boxes for pedestrians, providing 100 ms of historical event data. To address the class imbalance, the dataset uses under-sampling for the car class and over-sampling through horizontal flip data augmentation. The challenges presented by both datasets differ because of the variations in object sizes, shapes, and background complexity. Firstly, the MS-COCO dataset features objects with high variability in appearance and context, making it particularly well-suited for testing detection algorithms. Secondly, the Automotive Gen1 dataset specializes in automotive scenes, offering variety and dynamics that are important for object detection. To compare the results of the proposed approach with existing solutions for object detection using SNNs, we evaluated its performance against the best-performing SNNs. This evaluation demonstrated the relevance and worth of our proposed approach when dealing with the diverse and complex characteristics of the datasets.

### 4.2 Implementation details

For the experiments, the BIF neurons reset value $V_{reset}$, the membrane time constant *t*, the threshold $V_{t}h$, and the coefficient α were set to 0, 0.25, 0.5, and 1, respectively. We used an NVIDIA RTX3090 GPU with the SGD optimizer and a learning rate of $1{\text{e}}^{-2.}$ for training the models. The network underwent 300 epochs of training on the COCO dataset and 100 epochs on the Gen1 dataset, using batch sizes of 32 and 128, respectively.

**Performance Metrics:** Two main evaluation metrics—*mAP* & firing rate—are used to quantify the performance of object detectors. The *mAP* metric measures a detector’s ability to minimize false positives and their confidence scores while accurately localizing and classifying each item in a dataset. For object detection tasks, mAP@0.5 is the primary evaluation metric. The firing rate is crucial for assessing SNNs and refers to the average ratio of the total number of neuron spikes to the number of neurons across all time steps. On specialized hardware, computations occur only when spikes are released, making SNNs with lower firing rates potentially more energy-efficient.

### 4.3 Experimental results

In this section, we conduct a comparative analysis of the performance of SNN-based object detectors relative to various hybrid models. This evaluation includes new object detectors applied to the MS-COCO and Automotive GEN1 datasets. It is crucial to emphasize that the remaining models are similar to the SNN, having undergone a CNN-to-SNN conversion process followed by fine-tuning. Performance metrics, specifically mAP@0.5, for the baseline models trained on the COCO-source-train dataset and evaluated on these benchmark datasets, are detailed in Tables 1 and 2.

**Table 1:** Comparison of object detection models on the MS-COCO Dataset.

Model Name	Detection Head	Params	mAP@0.5 (%)	Infer (ms)
SNN-ResNet-18	YOLOv3	25.6M	38.2	55
SNN-ResNet-50	SSD	34.0M	40.7	61
Tiny-Yolo	Yolov3	-	25.8	35
SNN-ResNet-101	Faster R-CNN	44.5M	42.1	64
SNN-MobileNetV2	YOLOv3-Tiny	3.4M	33.5	45
SNN-DenseNet-121	RetinaNet	8.0M	39.8	59
SNN-EfficientNet	YOLOv4	7.5M	38.7	53
SNN-VGG16	SSD	138.0M	35.9	68
SNN-AlexNet	Faster R-CNN	60.0M	34.2	60
SNN-InceptionV3	RetinaNet	23.9M	41.3	66
EMS-Yolo(ResNet-34)	YOLOv3-Tiny	26.9M*	33.5*	-
**Proposed (ResNet34)**	**SSD**	**32M**	**47.6**	**36**

Abrivations are refered as: *Params (Parameters), *Infer (Inference Time)

**Table 2:** Comparison results on the automotive GEN1 dataset with best-performing models.

Models	Detection	Params	mAP@0.5	F.R. %	Infer (ms)
Spiking-YOLOv3	YOLO	61M	45.3	12	45
Spiking-Faster R-CNN (ResNet-50)	Fast R-CNN	50M	48.7	11	50
Spiking-SSD	SSD	34M	41.5	10	65
Spiking-YOLOv4	YOLO	64M	47.8	12	48
Spiking-RetinaNet	RetinaNet	40M	44.2	11	52
Spiking-CenterNet	CenterNet	35M	43.3	15	67
DSR-Fast R-CNN	RPN + Fast R-CNN	47M	49.1	11	53
Spiking-EfficientDet	EfficientDet	33M	42.7	10	39
VGG-11	SSD	12.6M	17.4	9	22
SEW-ResNet-50	YOLO	23M	43.1*	30*	40*
EMS-YOLO (ResNet-34)	YOLOv3	14M	50.1	27	40
**Proposed**	**SSD**	**23M**	**59.1**	**21**	**61**

Abrivations are refered as: *Params (Parameters), F.R. (Frame Rate), *Infer (Inference Time)

The proposed architecture ensures optimal spike-based encoding using bistable neurons. Membrane potential $V_{X,i,t}$ determines firing behavior, ensuring neurons transition efficiently between accumulation and activation phases. Spiking events occur based on threshold crossing, governed by Eq (6).This regulated activation mechanism allows low firing rates without compromising complex feature retention, as demonstrated in performance comparisons Tables 1 and 2.

The results presented in Table 1 highlight the performance of the proposed method compared to various selected models on the MS-COCO dataset. The table provides a comprehensive evaluation based on several critical metrics, including detection head type, model parameters, mAP@0.5, and inference time. The proposed method shows a significant advantage across these parameters. The proposed method utilizes the SSD’s detection head and is characterized by a parameter count of 32 million. Although this count is higher than that of some lightweight models like SNN-MobileNetV2 and SNN-DenseNet-121, it is significantly lower than more complex models such as SNN-VGG16 and SNN-AlexNet. The EMS-YOLO model [64], incorporating ResNet-34, demonstrates slightly higher accuracy; however, its parameters and inference time have not been disclosed, making it challenging to directly compare its performance with that of the proposed model. This moderate parameter size balances model complexity with computational efficiency. The proposed method achieves an impressive *mAP* of 47.6%, the highest among all listed models.

Notably, the proposed method outperforms several leading models, such as SNN-ResNet-101 (42.1%) and SNN-InceptionV3 (41.3%). This demonstrates that the proposed method not only excels in detection accuracy but also maintains competitive performance relative to more complex models.

In terms of inference time, the proposed method has a latency of 36 ms, which indicates that it requires less than 1000 time steps to achieve higher inference performance for object detection tasks. This performance surpasses that of several high-performing models, such as SNN-ResNet-101 (64 ms) and SNN-InceptionV3 (66 ms), which typically require 900-1000 time steps. This efficient inference time ensures that the proposed method achieves high detection accuracy while avoiding substantial computational overhead. Additionally, the proposed method exhibits a compelling balance between high accuracy and efficient performance. By utilizing the SSD’s detection head and maintaining a moderate parameter count, it achieves the highest mAP@0.5 among the evaluated models while ensuring competitive inference times.

The results presented in Table 2 for the Automotive GEN1 dataset provide a comprehensive assessment of the proposed method in comparison to other state-of-the-art models. The evaluation focuses on key performance metrics, including detection head type, *mAP*, parameter count, firing rate, and detection time. The proposed method demonstrates significant advantages across these metrics, confirming its superior performance and computational efficiency. The proposed model utilizes the SSD detection head with a parameter count of 23 million, which places it in a favorable position relative to other models. While the parameter count is higher than that of models such as Spiking-SSD (34M), it is notably lower than that of more complex architectures like Spiking-YOLOv3 (61M) and Spiking-YOLOv4 (64M). This balanced parameter count ensures that the model maintains a low computational burden while still achieving high accuracy. In particular, the moderate size of the model allows it to strike an optimal trade-off between model complexity and operational efficiency, positioning it as an ideal solution for resource-constrained environments. The proposed method achieves an exceptional *mAP* of 59.1%, the highest among all the models evaluated in the table. This result significantly outperforms other leading models, including EMS-YOLO (50.1%), Spiking-Faster R-CNN (48.7%), and Spiking-YOLOv3 (45.3%). The substantial improvement in *mAP* underscores the superior object detection capability of the proposed method, demonstrating its effectiveness in accurately identifying and localizing objects on the Automotive GEN1 dataset. This high detection accuracy is critical in practical applications where precise object localization and classification are essential. Additionally, the proposed model achieves a firing rate of 21%, which is the lowest among the models compared. For example, Spiking-YOLOv3 operates at a firing rate of 12%, while Spiking-YOLOv4 operates at 12%. The lower firing rate of the proposed method reflects a more efficient use of neural resources, leading to reduced computational overhead and lower energy consumption during inference. This improvement is particularly advantageous for deployment in low-power settings, where energy efficiency is a key consideration.

In terms of detection speed, the proposed method demonstrates an inference time of 61 ms per frame, making it a reasonable model among those evaluated. This is a significant improvement over other models, such as Spiking-Faster R-CNN (50 ms) and Spiking-RetinaNet (52 ms), both of which exhibit substantially higher inference times. The reduced detection time ensures that the proposed method is well-suited for real-time applications, where rapid decision-making is critical. This is a substantial improvement compared to the best-performing models, such as the EMS-YOLO (ResNet-34) (mAP: 50.1) and Spiking-Faster R-CNN (ResNet-50) (mAP: 48.7). The following aspects highlight why our approach is superior:

**Higher detection accuracy:** Our model outperforms other models like Spiking-YOLOv3 and Spiking-SSD in mAP@0.5 (59.1) due to its unique architecture design, which combines high accuracy and computational efficiency, and a reduction in parameters to 23M.

**Lower firing rate with higher accuracy:** The model maintains a balanced firing rate of just 21%—substantially lower than Spiking-YOLOv3 and Spiking-SSD—while still achieving superior mAP performance. This optimized firing rate ensures energy efficiency without compromising accuracy, resulting in low power consumption and high performance.

**Superior Efficiency in Time:** Our model has the inference speed of 61 ms per frame, outperforming other models like Spiking-YOLOv3, Spiking-SSD, and complex models like Spiking-Faster R-CNN. This low latency is crucial for real-time applications in autonomous vehicles and automotive systems, ensuring rapid inference while maintaining high accuracy.

**Parameter Efficiency:** The model, with 32/23 million parameters, outperforms other models like Spiking-YOLOv4 and Spiking-YOLOv3, resulting in lower memory and computational resource requirements, making it ideal for resource-constrained environments like automotive embedded systems.

While models like Spiking-YOLOv4 and Spiking-Faster R-CNN (ResNet-50) offer strong performance, their heavier architectures hinder efficiency. In contrast, our parameter-efficient design achieves optimal accuracy, speed, and power consumption, ensuring scalability for resource-constrained devices. With superior mAP, lower firing rates, and faster detection times, our method outperforms state-of-the-art models, making it an ideal choice for real-time automotive applications. These results highlight its technical superiority and practical viability, solidifying its role in advanced object detection.

#### 4.3.1 The effect of bistable neurons.

To evaluate the impact of bistable neurons in the proposed architecture (see Fig 3), we conducted experiments using single and double bistable neurons. The results, summarized in Table 3, reveal key differences between single and double bistable neuron configurations in terms of mean average precision (mAP@0.5) and time steps required for optimal inference. The single bistable neuron model achieved a mAP@0.5 of 47.6%, demonstrating superior object detection accuracy. This high precision stems from the neuron’s ability to encode temporal patterns effectively while minimizing spike redundancy. The bistable mechanism enhances stability by alternating between spike and non-spike states, allowing neurons to accumulate relevant information before firing. In terms of latency, this configuration maintained an efficient inference time of 36 ms, which corresponds to approximately 900–1000 time steps for the network to converge on a stable detection. The reduced number of spikes ensures that the computational cost remains low while maximizing temporal information density. This structure minimizes energy consumption and synaptic overhead, making it an optimal choice for real-time applications where rapid object detection is required. In contrast, the double bistable neuron model exhibited a lower mAP@0.5 of 43.2%, indicating a slight performance degradation in object detection accuracy. While additional bistable neurons improve temporal feature accumulation, they also introduce phase synchronization challenges. The extended inter-neuron communication in deeper layers can cause spike propagation misalignment, leading to less effective feature extraction.

**Table 3:** The results of adding neurons in the proposed SNN architecture using the MS-COCO dataset.

	Detection Head	mAP@0.5%	Inference Time (ms)
Single Bistable Neuron	SSD	47.6	36
Double Bistable Neuron	SSD	43.2	47

Our experimental results further validate the selection of T = 1000 as the optimal temporal window size for BSNN-based object detection. Table 1 (MS-COCO dataset) confirms that with T = 1000, our model achieves a mAP@0.5 of 47.6%, outperforming converted SNN architectures. Similarly, Table 2 (Automotive GEN1 dataset) supports our approach, showing a mAP@0.5 of 59.1% with an inference time of 6.1 ms per frame, proving low-latency efficiency while preserving temporal feature retention. Furthermore, results in Table 4 indicate that a single bistable neuron configuration achieves optimal accuracy while reducing inference latency to 36 ms. Comparatively, increasing neuron density introduces phase synchronization challenges without significant accuracy improvement. These findings confirm that T = 1000 enables efficient spike transmission, minimizing computational overhead while ensuring stable object detection performance.

**Table 4:** The results of adding IF/LIF or BIF Neurons in the proposed SNN architecture using the MS-COCO dataset.

	Detection Head	mAP@0.5%	Inference Time (ms)
IF Neuron	SSD	48.2	74
LIF Neuron	SSD	43.8	58
BIF Neuron	SSD	47.6	36

Moreover, the inference time increased to 47 ms, approximately 1200-1300 time steps, due to the additional neuron requiring longer periods for optimal firing synchronization. The accumulation of spikes over an extended interval results in a higher computational burden, which is detrimental to real-time efficiency. While the double neuron setup helps regulate spike precision, its higher latency makes it less suitable for time-sensitive detection tasks. The comparative results indicate that the single bistable neuron framework provides a more efficient trade-off between precision and latency. Given that object detection relies on fast response times and high detection accuracy, the increased complexity introduced by the double bistable neuron approach does not justify the marginal benefits in temporal stabilization. While double bistable neurons may improve information retention in certain scenarios, the increased latency makes them less suitable for time-sensitive tasks. Therefore, the single bistable neuron configuration strikes the best balance between accuracy, energy efficiency, and inference speed.

The selection of spiking neuron models significantly influences the efficiency, computational complexity, and detection performance of the proposed architecture. To systematically evaluate how our architecture performs in object detection tasks, we experimented with three distinct spiking neurons: Integrate-and-Fire (IF) neurons, Leaky Integrate-and-Fire (LIF) neurons, and Bistable Integrate-and-Fire (BIF) neurons. Their comparative results, presented in Table 4, highlight key trade-offs in detection accuracy (mAP@0.5), inference time, and computational efficiency.

The IF neuron is the simplest spiking neuron model, characterized by its ability to accumulate membrane potential until a threshold is reached, after which it generates a spike and resets. In our implementation, this neuron achieved the highest mAP@0.5 of 48.2% among the three tested neuron models, demonstrating superior object detection accuracy. However, this high accuracy comes at a cost—inference latency. The IF-based architecture required 74 ms for object detection, which is significantly higher than both the LIF and BIF models. This extended inference time can be attributed to spike rate saturation, where neurons accumulate excessive charge before firing, increasing the number of required simulation steps. As seen in prior analyses (Figs 2 and 3), excessive firing in deep layers contributes to temporal misalignment, impacting network stability and energy efficiency.

LIF neurons introduce an additional leakage term, which enables the gradual dissipation of accumulated charge over time. This mechanism facilitates more stable spiking dynamics, reducing excessive charge build-up and enhancing temporal efficiency. Despite this improvement, the LIF-based architecture exhibited a lower mAP@0.5 of 43.8%, suggesting that the leakage mechanism, while improving convergence speed, leads to potential information loss in feature retention. The inference time was moderately reduced to 58 ms, illustrating an improvement in efficiency compared to the IF neuron. From a computational standpoint, the introduction of leakage improves spike frequency regulation, reducing synaptic redundancy. However, its lower detection accuracy and suboptimal inference speed suggest that LIF neurons, while beneficial in energy conservation, may not be ideal for high-precision object detection tasks.

The BIF neuron framework, as introduced in Sect 3.4 and illustrated in Fig 3, operates under bistable dynamics, alternating between spiking and non-spiking phases to enhance temporal encoding. This bistability mechanism improves information retention while significantly reducing spike overhead. Compared to IF and LIF neurons, the BIF-based architecture achieved a competitive mAP@0.5 of 47.6%, demonstrating strong detection accuracy. Most notably, it exhibited the lowest inference time of 36 ms, enabling efficient real-time performance. This rapid processing speed is attributed to phase-aligned accumulation, wherein non-spiking periods allow neurons to retain information while minimizing computational redundancy.

Furthermore, as shown in Table 3, the single BIF neuron configuration achieves superior performance in terms of accuracy and latency compared to double neuron architectures, reinforcing that bistable encoding is optimal for time-sensitive applications. Considering the findings from Table 4, BIF neurons emerge as the most effective spiking neuron model for object detection in our architecture, as they offer optimal performance with reduced temporal steps, preserving spatial-temporal features without incurring excessive computational delays.

### 4.4 Future work

BIF neurons tackle key challenges in spiking neural networks, notably reset-related information loss and inefficient spike encoding. Their bistability mechanism enhances signal propagation, reduces firing rates, and improves energy efficiency, making them ideal for large-scale computations. Beyond object detection, BIF neurons hold potential in semantic segmentation, improving spatial retention and encoding, and in generative models, ensuring stable signal transmission for high-quality data synthesis. Their compatibility with deep architectures like ResNet makes them scalable across AI applications. Future research should explore adversarial conditions, occlusions, and perturbations to validate their generalization capabilities, further refining their use across segmentation, neural synthesis, and diverse network configurations.

## 5 Conclusion

This study thoroughly examined the factors contributing to performance degradation and extended temporal delays associated with the CNN-to-SNN conversion process. It was found that the instantaneous response of neurons to incoming current in converted SNNs is inherently unreliable, leading to significant instability. This instability results in the firing rates in deeper layers failing to accurately approximate the activation values observed in CNNs, particularly in object detection tasks. To address these issues, we propose a novel network architecture that integrates BIF neurons with ResNet capabilities specifically tailored for object detection. This architecture is enhanced with the SSD’s detection head, enabling effective utilization of the network for object detection applications. Empirical results demonstrate that the proposed method achieves object detection within reduced temporal intervals, with detection times of 36ms and 61ms on the MS-COCO and Automotive GEN1 datasets, respectively. Additionally, the proposed network achieved outstanding accuracy with mAP@0.5 scores of 47.6% and 59.1% on the MS-COCO and Automotive GEN1 datasets, respectively. Future research will focus on deploying these SNNs on low-power hardware to facilitate power-efficient embedded applications.
